# A Novel Sparse Cellular Labeling System with Tunable Gradients and Long‐Term Stability

**DOI:** 10.1002/advs.77438

**Published:** 2026-08-30

**Authors:** Xiaomei Tang, Zhenye Hou, Li Lu, Yige Song, Changdong Chai, Lanfang Li, Yuhang Shen, Shuo Wang, Ya Zhao, Jinyu Zeng, Yiqing Guo, Fuliang Jiang, Zhigao Xiang, Hao Li, Aodi He, Bing Zhang, Youming Lu, Xinyan Li

**Affiliations:** ^1^ School of Basic Medicine Tongji Medical College Huazhong University of Science and Technology Wuhan China; ^2^ Innovation Center of Brain Medical Sciences Ministry of Education of the People's Republic of China Wuhan China; ^3^ Department of Pathology The Central Hospital of Wuhan Tongji Medical College Huazhong University of Science and Technology Wuhan China; ^4^ Department of Plastic Surgery Union Hospital Tongji Medical College Huazhong University of Science and Technology Wuhan China; ^5^ Department of Neurosurgery Tongji Hospital of Tongji Medical College Huazhong University of Science and Technology Wuhan China; ^6^ Department of Pathology Renmin Hospital of Wuhan University Wuhan China

**Keywords:** competitive recombination, long‐term stability, sparse labeling, tunable gradients

## Abstract

The dense packing of cells within tissues poses challenges for studying cellular properties. Sparse labeling, genetically targeting a small subset of densely distributed cells, provides a powerful approach to investigate cellular morphology, connectivity, dynamics, and functions, especially in neuroscience. However, current sparse labeling methods are generally restricted to fixed labeling densities and often exhibit a decline in sparsity over time. In this study, by maximizing the utility of commonly used recombinases and the relational recognition sites, we constructed a versatile sparse labeling system, termed Tri‐M (Multi‐recombinase, Multi‐recognition site, and Multi‐nested), built upon competitive recombination. The Tri‐M system, which integrates transgenic mice with viral injection, enables tunable and graded sparse labeling of specific cell types within specific brain regions with long‐term stability. This system significantly enhances the ability to track, analyze, and manipulate cells within tissues characterized by dense cellular packing, from single cells to populations.

## Graphical abstract

The Tri‐M (Multi‐recombinase, Multi‐recognition site, and Multi‐nested) sparse cellular labeling system, built upon competitive recombination, integrates transgenic mice with viral injection to achieve tunable and long‐term stable sparse labeling of specific cell types with region selectivity, from sparse populations to individual cells, facilitating more precise investigation of cellular morphology, connectivity, and functions.

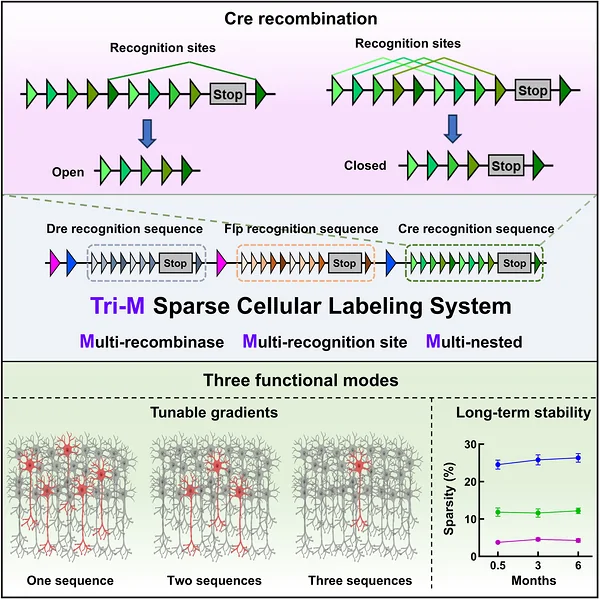

## 1 Introduction

The cell type‐specific labeling technology mediated by restrictive recombinases provides a critical tool for dissecting the biological functions of the labeled cells [1, 2]. However, the classic “all‐or‐none” recombinase‐mediated labeling strategies possess inherent limitations: population‐level studies fail to reveal the heterogeneity of individual cells [3, 4]; meanwhile, the dense packing of cells within tissues also restricts the visualization of cellular morphology and its connectivity [5, 6]. Therefore, achieving sparse labeling of specific cell types, a strategy whereby only a minimal fraction of cells within a population is labeled, is of significant importance for in‐depth analysis of cellular properties. This demand is particularly prominent in neuroscience, where, beyond functional properties, the morphological features of cells, as well as their long‐range axonal projections and connections, also require systematic investigation [7, 8].

Current sparse labeling strategies primarily fall into two categories. The first category is based on transgenic mouse crosses, achieving sparse labeling through stochastic recombination‐mediated gene expression, as represented by systems such as MADM [9], SPARC [10], and MORF [11]. These strategies enable long‐term stable sparsity, but the labeling density is relatively fixed and lacks region selectivity, making it difficult to meet the requirements of density modulation or region‐specific targeting. The second category relies on stereotaxic injection of viral vectors, achieving sparse labeling through low‐probability burst expression, with representative techniques including Semliki Forest virus systems [12], dual‐AAV sparse labeling systems [13], Supernova [14], and STARS [15]. Despite enabling region‐specific targeting, these strategies exhibit inconsistent sparsity owing to progressive accumulation of labeled cells over time, thereby limiting their applicability for long‐term studies of cellular properties. To overcome the limitations of the existing strategies, establishing a labeling system that combines regional targeting, modifiable sparsity, and long‐term stability is of great importance for advancing the study of cellular properties, including at the single‐cell level.

Therefore, based on the mechanism of competitive recombination within restrictive recombinase systems, this study developed a novel Tri‐M (Multi‐recombinase, Multi‐recognition site, and Multi‐nested) sparse cell labeling system. The core component of this system, the Tri‐M sequence, is designed with nested pairs of recognition sites for Dre (D6 recombinase) [16], Flp (Flippase) [17], Cre (Cre recombinase) [18], as well as the orthogonal Cre variants VCre and SCre [19]. Building on this, we established the Tri‐M1 transgenic mouse line. By crossing with Cre driver lines and combining with stereotactic injection of viral vectors expressing different recombinases, we achieved labeling at three graded sparsity levels (approximately 25%, 10%, and 4%) in specific brain regions and cell types, with stable maintenance of sparsity for extended periods (6 months). To achieve even higher sparsity down to the single‐cell level, we generated the second‐generation (Tri‐M2) transgenic mouse line, which incorporates a closed positive feedback loop involving leaky tTA expression from the tetracycline‐responsive element promoter (pTRE) [14, 20], enabling a sparsity level lower than 1‰. In summary, the Tri‐M system established in this study is a comprehensive sparse labeling system integrating transgenic mouse models and viral tools. This system combines the advantages of existing sparse labeling strategies, enabling region selectivity, tunable gradients, and long‐term stable sparse labeling for specific cell types. As such, it offers a powerful technological platform for investigating cellular properties, including morphology, connectivity, and functions, across tunable levels of sparsity: from sparse populations to individual cells.

## 2 Results

### 2.1 Principle of the Tri‐M Sequence

To achieve sparse and bright cell labeling, we developed a Tri‐M sparse cellular labeling system that integrates transgenic mouse models and viral tools. The Tri‐M sequence, a multi‐level nested cassette consisting of tandem recognition sequences for Dre, Flp, and Cre recombinases, serves as the core component of this system (Figure 1A and Table S1). Each recognition sequence contains multiple nested pairs of recognition sites for one recombinase, with a transcriptional termination cassette (Stop) inserted upstream of the final site (Figure 1B, Figure S1A,D, and Table S2). Each Stop cassette is a composite multi‑copy polyadenylation termination module assembled from tandem arrays of commonly used poly (A) signals, including SV40 poly (A) signal [21, 22], bGH poly (A) signal [23], PGK poly (A) signal [24], β‐globin poly (A) signal [25], and pause site [26]. The design principle is as follows: only when the recombinase reacts with the final pair of recognition sites is the Stop cassette excised (open), allowing the downstream sequence to be transcribed (Figure 1C and Figure S1B,E); conversely, if recombination occurs at any other pair of sites, the Stop cassette is retained (closed), and the downstream sequence cannot be transcribed (Figure 1D and Figure S1C,F). This mechanism achieves sparse labeling by ensuring that the probability of a recognition sequence remaining closed is substantially higher than its probability of being open. Furthermore, regardless of which pair of sites undergoes recombination, the residual sites left after excision cannot form a pair. This ensures the uniqueness and permanence of the resulting sequence, which is the critical foundation for the Tri‐M system to maintain a stable probability of sparse labeling over extended time scales.

**FIGURE 1:**
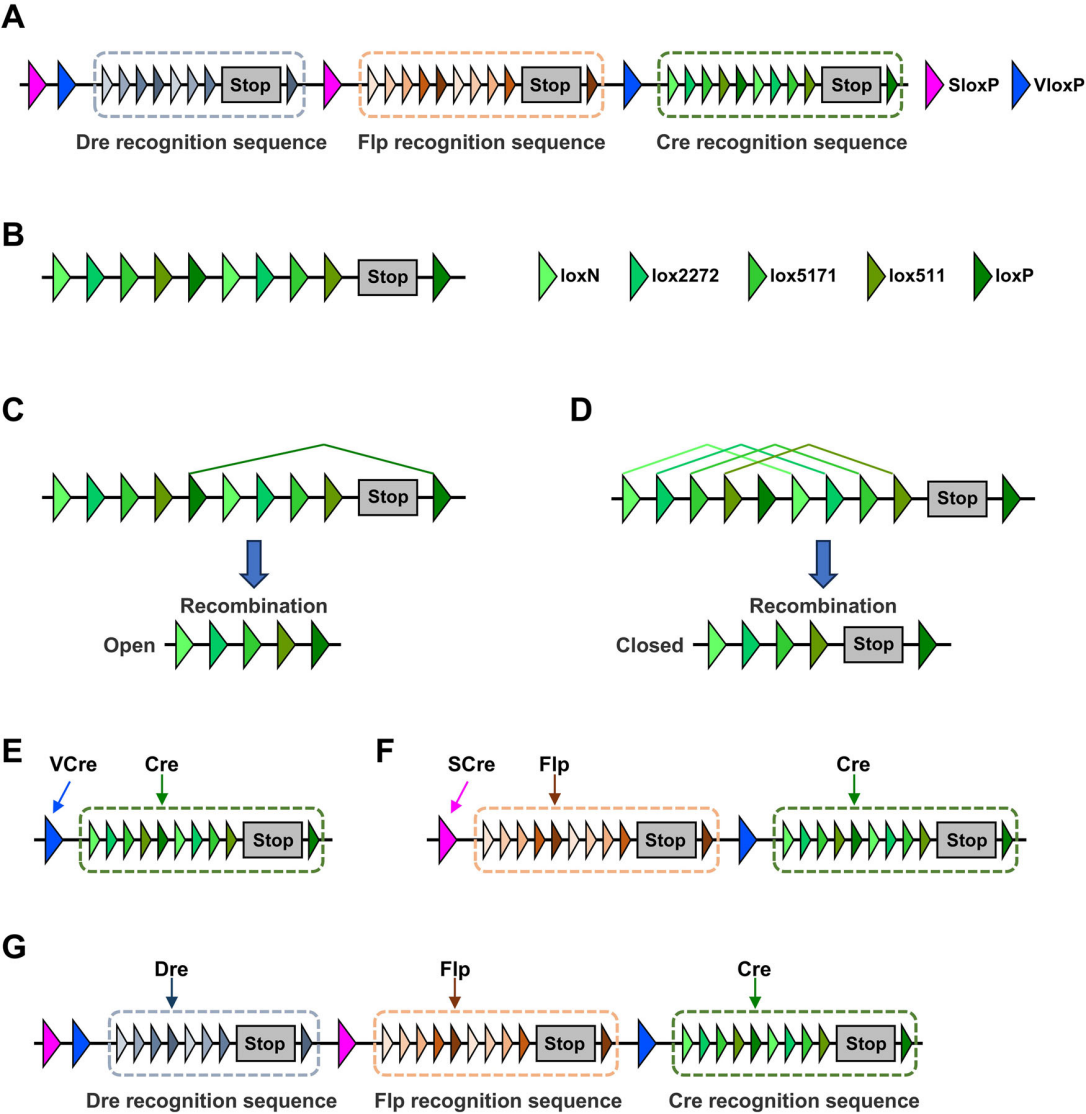
Construction and functional principle of the Tri‐M sequence. (A) Schematic diagram of the Tri‐M sequence construct. (B–D) Schematic diagrams of the Cre recognition sequence (B) and its open (C) and closed (D) configurations following recombination. (E–G) Three functional modes of the Tri‐M sequence: low (E), medium (F), and high (G) sparsity, achieved through recombination with different recombinases.

To achieve tunable graded sparse labeling, the recognition sites SloxP and VloxP for SCre and VCre were inserted in a nested configuration within the Dre, Flp, and Cre recognition sequence (Figure 1A). By controlling the number of recognition sequences involved, the open probability of the Tri‐M sequence can be modulated in three modes. Theoretical analysis indicates that when VCre and Cre recombinases are introduced together, only the Cre recognition sequence is functional, resulting in a high open probability (low sparsity) of the Tri‐M sequence (Figure 1E). When SCre, Flp, and Cre recombinases are introduced together, both Flp and Cre recognition sequences are engaged, leading to a medium open probability (medium sparsity) (Figure 1F). When Dre, Flp, and Cre recombinases are introduced together, all three recognition sequences are simultaneously involved, reducing the open probability to the lowest level (high sparsity) (Figure 1G). Given that Cre driver mouse lines currently represent the most effective tool for achieving cell‐type‐specific labeling [27, 28, 29], we positioned the Cre recognition sequence at the terminus of the Tri‐M sequence. This configuration ensures that all three sparsity levels require Cre recombinase, thereby allowing flexible use of the extensive collection of existing Cre driver lines.

### 2.2 Graded Sparse Labeling and Morphological Visualization of Specific Neuronal Types Using the Tri‐M System

To assess the feasibility of the Tri‐M system for sparse labeling, we generated a CAG promoter (pCAG)‐driven construct containing the Tri‐M sequence, tdTomato‐P2A‐tTA2, and WPRE, and established a transgenic mouse model (named Tri‐M1) by targeted insertion into the *Rosa26* locus [30]. This design ensures that tdTomato is expressed only in cells where the Tri‐M sequence is open (Figure 2A). Previous studies have demonstrated that cryptic splice sites within the Stop cassettes can mediate cryptic mRNA splicing [31, 32]. To assess whether such recombinase‑independent labeling occurs in the Tri‑M system, we examined brain sections from recombinase‑negative Tri‑M1 mice, and no red fluorescent cells were observed throughout the brain (Figure S2A). To validate the capability of the Tri‐M system for achieving graded sparse labeling in neurons, we first chose to examine excitatory neurons in the cerebral cortex. To this end, Tri‐M1 mice were crossed with NEX‐Cre mice, a line in which Cre recombinase is specifically expressed in excitatory neurons of the cerebral cortex and hippocampus. Given that the ventral tegmental area (VTA) receives cortical input specifically from layer V (L5) excitatory neurons of the cerebral cortex [33], we stereotaxically injected a Cre‐dependent, retrogradely transported recombinant adeno‐associated virus (rAAV2‐retro‐DIO) carrying recombinases (VCre, Flp/SCre, or Flp/Dre) and EGFP into the VTA of NEX‐Cre::Tri‐M1 mice. This approach enabled graded sparse labeling of L5 excitatory neurons in the cerebral cortex (Figure 2B). After 15 days, these injections resulted in EGFP‐positive neurons (EGFP^+^) densely located in L5 throughout the cerebral cortex, and only a small portion of EGFP^+^ neurons expressed tdTomato (tdTomato^+^), which were sparsely distributed. The tdTomato^+^ neurons and their long‐range axonal projections in these three groups (VCre, Flp/SCre, and Flp/Dre) exhibited a clear sparsity difference. Quantitative analysis revealed that the sparsity level of the Tri‐M system, defined as the percentage of tdTomato^+^ neurons among EGFP^+^ neurons, closely aligned with theoretical gradations: the VCre/Cre combination yielded high labeling density (24.52% ± 1.2%), followed by the SCre/Flp/Cre combination with moderate density (11.84% ± 1.1%), and the Dre/Flp/Cre combination with low density (3.75% ± 0.37%) (Figure 2C,G and Figure S2B,C). Driven by the WPRE enhancer [34, 35], tdTomato achieved robust expression, enabling clear visualization of neuronal cell bodies, axons, and dendritic spines (Figure 2D). Notably, the unique and irreversible open/closed features of the Tri‐M sequence ensured that each sparsity level remained stable at 3 and 6 months after injection (Figure 2E–G).

**FIGURE 2:**
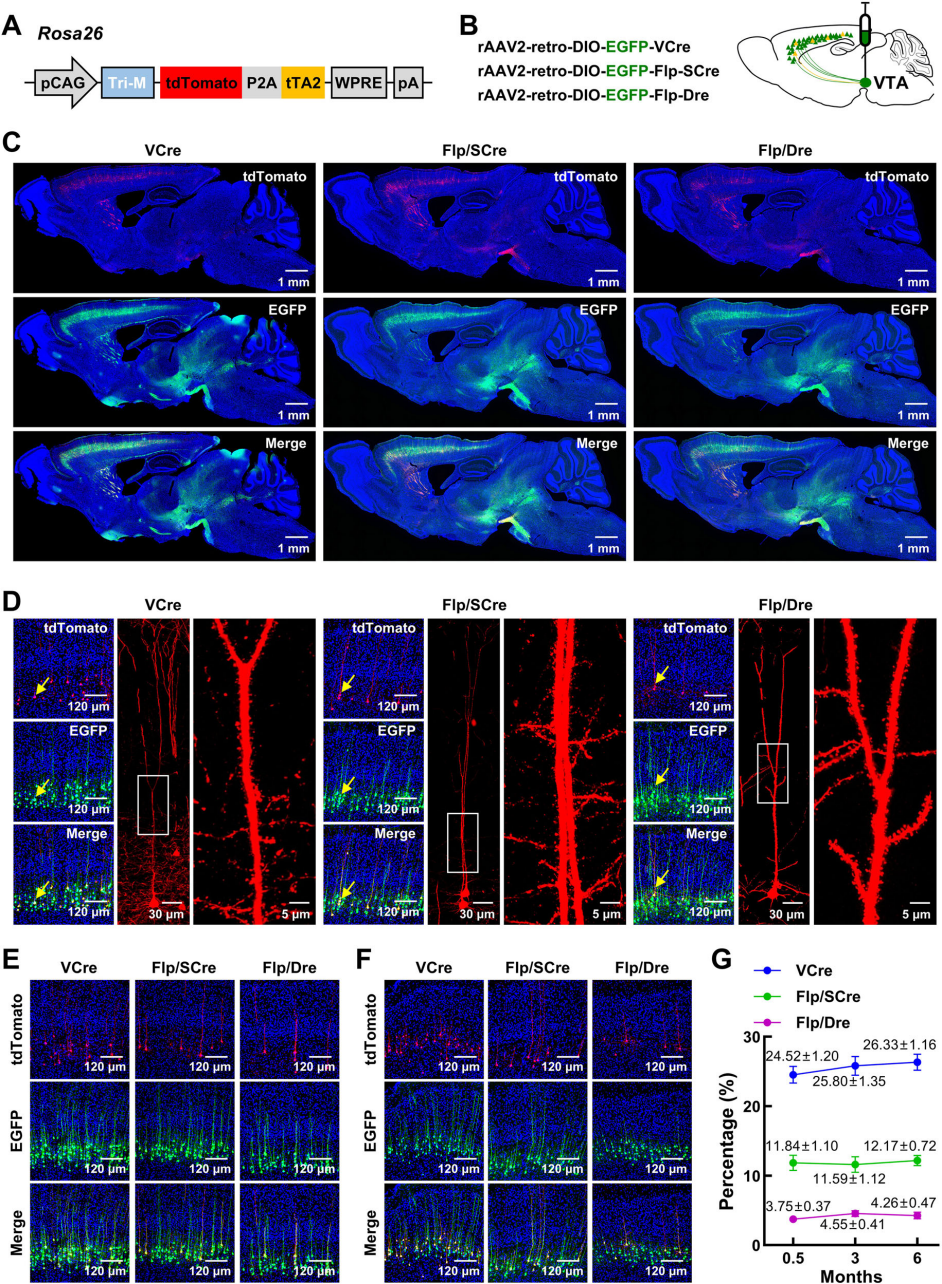
Graded sparse labeling of excitatory neurons in L5 of the cerebral cortex. (A) Schematic diagram of the construct inserted into the *Rosa26* locus of the Tri‐M1 mouse. (B) Schematic of viral injection for graded sparse labeling of L5 excitatory neurons in the cerebral cortex using NEX‐Cre::Tri‐M1 mice. (C, D) Images showing graded sparse labeling of excitatory neurons in L5 of the cerebral cortex 15 days after viral injection: (C) in whole‐brain sections; (D) in the cerebral cortex with higher‐magnification views of individual neurons and dendrites. (E, F) Images showing the cerebral cortex with graded sparse labeling of excitatory neurons in L5 3 months (E) and 6 months (F) after viral injection. (G) A line graph depicting labeling sparsity at different time points between viral injection and perfusion, corresponding to the three functional modes of the Tri‐M system. Data are presented as mean ± SEM (n = 5 mice per group).

To further evaluate the applicability of the Tri‐M system for labeling different neuronal types within different brain regions, we sparsely labeled excitatory granule cells in the dentate gyrus (DG), inhibitory PV (parvalbumin) neurons in the visual cortex, and cholinergic neurons in the basal forebrain, by injecting Cre‐dependent, locally infected adeno‐associated virus rAAVs (rAAV2/9‐DIO‑EGFP‐VCre, rAAV2/9‐DIO‑EGFP‐Flp‐SCre, or rAAV2/9‐DIO‑EGFP‐Flp‐Dre) into the Calb1‐Cre::Tri‐M1 (Figure 3A,D and Figure S2F), PV‐Cre::Tri‐M1 (Figure 3B,D and Figure S2D), and ChAT‐Cre::Tri‐M1 mice (Figure 3C,D and Figure S2E, G), respectively. Sparsity levels also varied in a graded manner depending on the choice of recombinases, and the morphology of the labeled cells was equally clearly visible.

**FIGURE 3:**
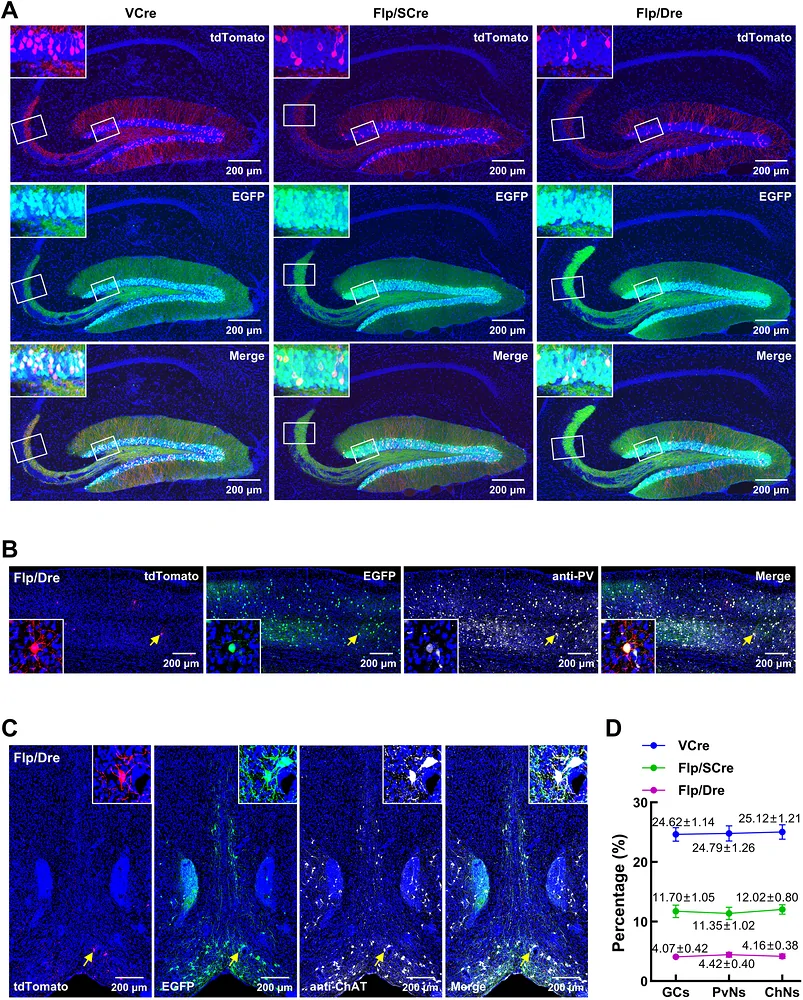
Graded sparse labeling of distinct neuronal types in different brain regions. (A) Images showing graded sparse labeling of granule cells in the dentate gyrus (DG). (B, C) Images showing sparse labeling of parvalbumin (PV)‐positive neurons in the visual cortex (B) and cholinergic neurons in the basal forebrain (C) with the high sparsity mode of the Tri‐M system. Labeling specificity was confirmed by immunostaining with anti‐PV and anti‐ChAT, respectively. (D) A line graph showing labeling sparsity for different neuronal types across the three functional modes of the Tri‐M system, assessed 15 days after viral injection. Data are presented as mean ± SEM (n = 5 mice per group).

### 2.3 Sparse Labeling of Dividing Astrocytes Using the Tri‑M System

Despite the unique and irreversible features of the Tri‐M sequence, heritability is also a key characteristic, meaning that the open/closed state of the Tri‐M sequence can be inherited by daughter cells. Mature neurons in the brain are non‑dividing and thus have no daughter cells [36, 37]; we sparsely labeled them using Cre mice commonly employed in neuronal labeling, as Cre is expressed during the neuronal maturation phase. For dividing cells [38, 39], however, the earlier the opening or closing of the Tri‐M sequence occurs, the more unstable the sparsity becomes. As an extreme example, if the Cre recognition sequence is recombined to a closed state in the oosperm by crossing with Cre mice, then the Cre recognition sequence in all cells of the resulting mouse will be closed, and consequently the Tri‐M sequence will also be closed, resulting in a labeling probability of 0 for the Tri‐M system [40]. Conversely, if the Cre recognition sequence remains open in the oosperm, the subsequent labeling probability of the Tri‐M system will be increased. Therefore, we crossed inducible Cre mice [41, 42] with Tri‐M1 mice and induced recombination with tamoxifen to limit the persistence of the recombined state of the Cre recognition sequence, thereby stabilizing the sparsity level. To this end, we crossed Aldh1l1‐CreERT2 mice, an inducible Cre line specifically expressed in astrocytes, with Tri‐M1 mice (Aldh1l1‐CreERT2::Tri‐M1), and injected rAAV2/9‐DIO‑EGFP‐VCre, rAAV2/9‐DIO‑EGFP‐Flp‐SCre, or rAAV2/9‐DIO‑EGFP‐Flp‐Dre into the CA1 region of the hippocampus. Tamoxifen was administered once daily for 4 days (from Day 2 to Day 5 after viral injection on Day 1), and the mice were perfused (Day 20) 15 days after the final tamoxifen injection (Figure 4A). Both EGFP and tdTomato were expressed in astrocytes (anti‑GFAP), and the graded sparsity levels were consistent with those previously observed in neurons (Figure 4B,C).

**FIGURE 4:**
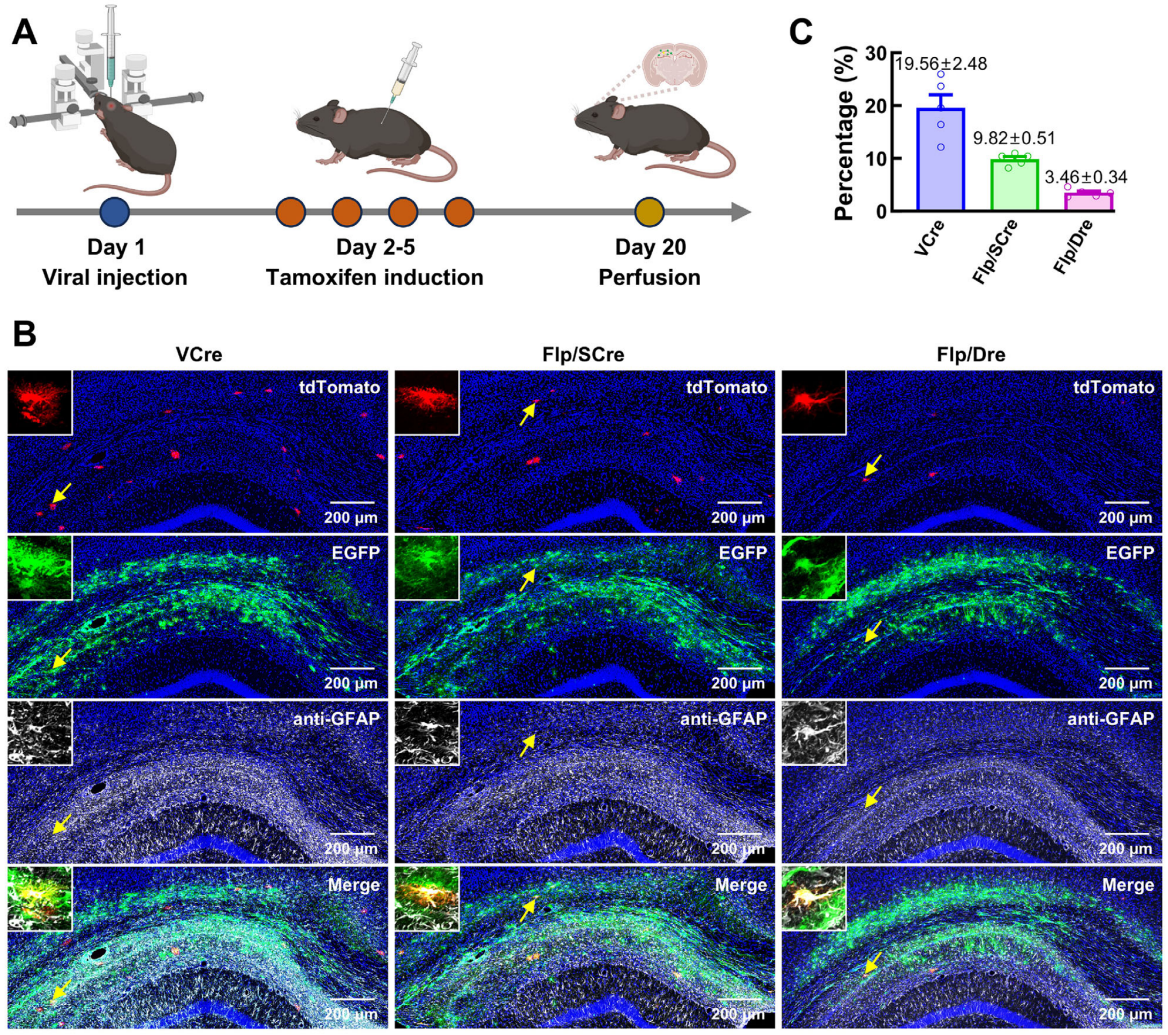
Graded sparse labeling of astrocytes. (A) Schematic of the experimental timeline, including viral injection, tamoxifen induction, and perfusion. (B, C) Images (B) and a bar graph (C) showing graded sparse labeling of astrocytes in the CA1 region of the hippocampus across the three functional modes of the Tri‐M system. Labeling specificity was confirmed by immunostaining with anti‐GFAP. Data are presented as mean ± SEM (n = 5 mice per group).

### 2.4 Expression of Functional Protein Elements and Versatile Applications of the Tri‑M Sparse Labeling System

The cellular functional analysis relying on “all‐or‐none” labeling has inherent limitations, as its interpretation is overly simplistic and binary, and may not accurately and comprehensively reflect the physiological functions of cells. Therefore, sparse labeling also holds significant value for investigating cellular functions. However, sparse labeling with fluorescent proteins allows only for the visualization of cellular morphology, and thus the in‐depth exploration of diverse physiological functions necessitates the introduction of functional elements. To address this, we introduced the tTA2 element downstream of tdTomato in the Tri‐M1 mouse (Figure 2A); this element can specifically activate the TRE promoter (pTRE). In this way, tdTomato and tTA2 are co‑expressed in sparsely labeled cells, enabling the introduction of desired protein elements in conjunction with the TRE promoter. On the basis of sparse labeling of L5 cortical neurons with low sparsity using VCre and Cre recombinases (Figure 2B–D), we respectively injected rAAV2‐retro‐DIO‐EBFP‐VCre and rAAV2/9‑pTRE‑ChR2‐EYFP into VTA and the sensory cortex of NEX‐Cre::Tri‐M1 mice to achieve specific expression of the light‑sensitive channel ChR2 and EYFP in sparsely tdTomato^+^ neurons within L5 of the sensory cortex (Figure 5A–C), thereby allowing these neurons to be specifically manipulated by blue light delivery (Figure 5D). Thus, using the Tri‐M system, functional protein elements can be introduced into sparsely labeled cells, enabling cellular manipulation and functional studies.

**FIGURE 5:**
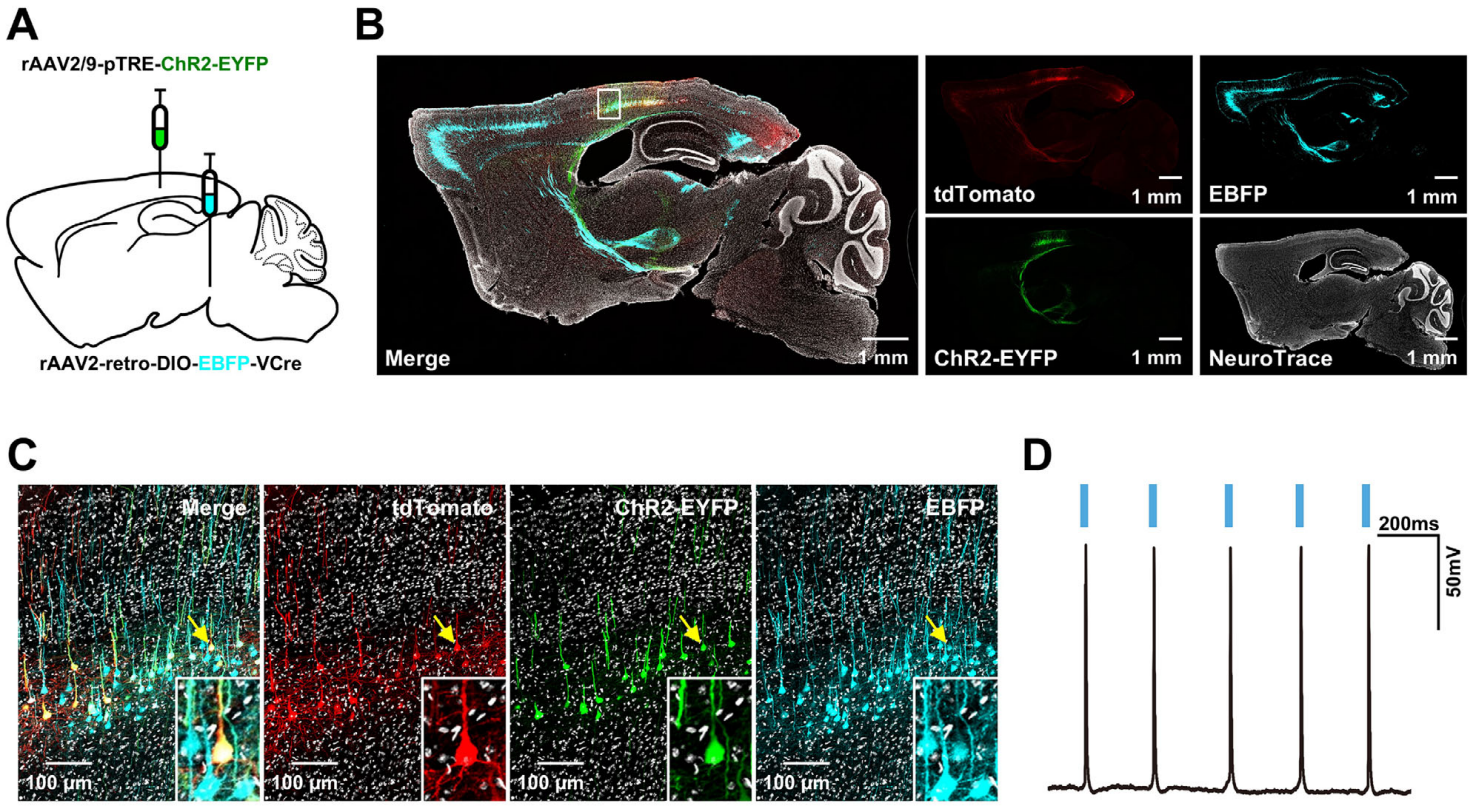
Cellular manipulation following sparse labeling with the Tri‐M system. (A‐C) Schematic of viral injection (A) and images showing specific labeling of L5 excitatory neurons in the sensory cortex with ChR2‐EYFP under the low sparsity mode of the Tri‐M system (B, whole‐brain section; C, magnified view of sensory cortex) 15 days after viral injection. (D) Evoked action potentials recorded from EYFP‐labeled neurons in L5 of the sensory cortex in response to blue light stimulation 15 days after viral injection.

In addition to enabling cellular manipulation, the Tri‑M sparse labeling system can advance research based on cellular imaging, such as in vivo imaging and spatial transcriptomics. Its sparsity minimizes the overlap of target cells and allows individual cells to be resolved with exceptional clarity. Furthermore, when combined with cellular lineage analysis and circuit tracing, this system enables more precise mapping of cell lineage and connectivity. Collectively, these features establish the Tri‐M system as a versatile and robust platform for comprehensive characterization of cellular properties.

### 2.5 Extending the Tri‐M System to Achieve Sparse Labeling at the Single‐Cell Level

To advance the research of cellular properties at the single‐cell level, we further optimized the sparse labeling system based on the Tri‐M system. By integrating the leaky expression property of the tetracycline‐responsive element promoter (pTRE) with design principles from the Supernova system [14], we created a novel Tri‐M2 transgenic mouse line, in which the inserted construct in the Tri‐M1 mouse was modified by replacing the pCAG with the pTRE2 (a modified version with lower leaky expression) and specifically inserted into the *TIGRE* locus [43] (Figure 6A). Similar to Tri‑M1 mice, recombinase‑negative Tri‑M2 mice also exhibited no red fluorescent cells throughout the brain (Figure S3A). In the Tri‐M2 mouse, leaky expression from pTRE leads to minimal tTA2 (along with tdTomato) production exclusively in a small subset of sparse cells where the Tri‐M sequence is open. This minimal tTA2 then activates pTRE in a closed positive feedback loop, resulting in robust expression of tdTomato and tTA2 (Figure 6B). We injected the same rAAVs in the same manner as in Figure 2 into the NEX‐Cre::Tri‐M2 mice to sparsely label L5 excitatory neurons in the cerebral cortex. Notably, the graded neuronal labeling densities (approximately 7‰, 3‰, and 0.7‰) were substantially lower than those observed in NEX‐Cre::Tri‐M1 mice, corresponding to considerably higher sparsity levels, with only a few tdTomato^+^ neurons—sometimes as few as a single cell—detectable throughout a sagittal brain section, thereby achieving sparse labeling at the single‐cell level (Figure 6C–E and Figure S3B–E). Moreover, the expression level of tdTomato and tTA2 is not a concern, given that the closed positive feedback pTRE‐tTA loop yields far higher expression than the classical CAG promoter [43].

**FIGURE 6:**
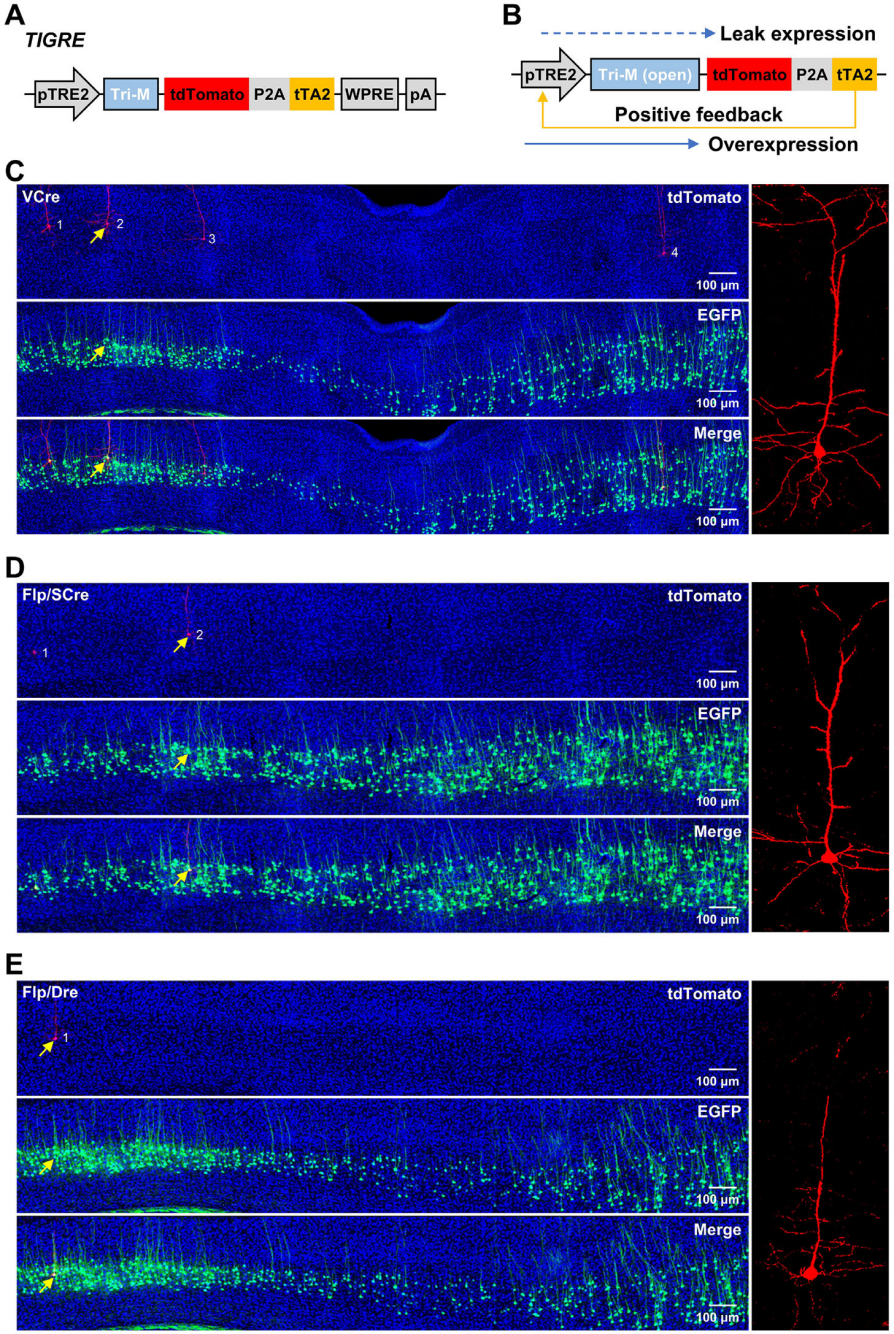
Graded sparse labeling of excitatory neurons in L5 of the cerebral cortex in Tri‐M2 mice. (A, B) Schematic diagram of the construct inserted into the *TIGRE* locus of the Tri‐M2 mouse (A) and its functional principle (B). (C‐E) Images showing graded sparse labeling of L5 excitatory neurons in the cerebral cortex and the higher‐magnification views of tdTomato‐labeled individual neurons across the three modes of the Tri‐M system in NEX‐Cre::Tri‐M2 mice 1 month after viral injection.

## 3 Conclusion

All recombinase recognition sites selected for the Tri‐M sequence are well‐characterized and highly specific; each site pair is recognized exclusively by its corresponding recombinase, and unmatched sites are in principle incapable of being recognized by recombinases. We acknowledge that off‐target recombination between non‐matching site pairs has been reported [44, 45], and the possibility of such misrecognition theoretically exists within the Tri‐M sequence. However, it is important to note that the probability of these misrecognition events is extremely low. Moreover, within each recombinase recognition sequence, which contains eight or ten recognition sites, misrecognition must occur at the final site located downstream of the Stop cassette to excise it and open the sequence. This requirement further reduces the potential impact of misrecognition on the Tri‐M sequence. Indeed, even under prolonged exposure to recombinases, the sparsity levels of cells labeled by the Tri‐M system did not significantly change (Figure 2G), indicating that the probability of misrecognition events that excise the Stop cassettes occurring at the unpaired sites remaining in closed recombinase recognition sequences is very low. Therefore, the Tri‐M system is a reliable, credible, and practical sparse labeling platform that combines region selectivity, tunable gradients, and long‐term stability for sparse labeling across diverse cell types.

Currently, Cre driver mice represent the most efficient tool for labeling specific cell types [27, 46]. Thus, we placed the Cre recognition sequence at the terminal position of the Tri‐M sequence to ensure that all three open modes of the Tri‐M sequence depend on Cre recombination, thereby facilitating straightforward integration with existing Cre driver lines. This design does not, however, preclude the use of Flp or Dre driver mice with the Tri‐M system; rather, it simply offers fewer sparsity options when these recombinase driver mice are employed. For example, Flp driver mice must be used in combination with Cre recombinase introduced via a viral vector, and two open modes (moderate and low labeling density) of the Tri‐M sequence can be chosen depending on whether the Dre recognition sequence is employed. Similarly, Dre driver mice must be used in combination with both Flp and Cre, resulting in only one open mode (low labeling density) of the Tri‐M sequence. Thus, both Flp and Dre driver mice remain fully compatible with the high sparsity (low labeling density) setting of the Tri‐M system, enabling high‐level sparse labeling when needed.

For cell‐type‐specific labeling, the recombinase recognition sequences allow perfect integration with Cre, Flp, or Dre driver lines to label defined populations (Figures 2 and 3), including dividing cell types that are challenging to stably label with other sparse labeling methods (Figure 4). Although our current demonstration is limited to neurons and astrocytes in the mouse brain, the Tri‐M system is readily applicable to other tissues and cell types throughout the body by using specific recombinase driver lines and site‐directed viral injection, and even to rats, dogs, and monkeys by establishing the species‐specific Tri‐M system. For cellular physiological and functional studies, tTA2 expression enables the use of Tet‐Off‑based viral vectors carrying functional elements [47], which, together with the sparsity of the Tri‑M system, allows more precise investigations of specific cells.

Compared to existing sparse labeling methods [48], the Tri‐M system exhibits remarkable extensibility. Its underlying principle relies on competitive recombination among recombinase recognition sites, constituting an expression control module (the Tri‐M sequence) that operates independently of promoter selection or expression regulation. This modular architecture provides flexible interfaces for compatibility with various viral vectors, promoter systems, and regulatory strategies, significantly expanding the potential applications of the Tri‐M system. Thus, the Tri‐M system can be readily combined with other sparse labeling strategies that do not conflict with its core mechanism. As exemplified by the Tri‐M2 mouse, derived from Tri‐M1 by integrating the closed positive feedback pTRE‐tTA loop based on pTRE leaky expression [14, 20], further enhancements in sparsity can be achieved to meet more stringent labeling demands. Moving forward, the Tri‐M system can be further combined with other labeling and expression methodologies, expanding the toolbox with additional labeling sparsity and functional elements to offer researchers broader experimental flexibility.

The Tri‐M2 mouse is characterized by pTRE leaky expression and the subsequent activation of the closed positive feedback pTRE‐tTA loop. However, as the duration between viral injection and perfusion increased, the density of labeled cells increased over time (Figure S3), whereas no such phenomenon was observed in the Tri‐M1 mouse lines (Figure 2G). This suggests that the probability of pTRE leaky expression increases over time, rather than an increase in the probability of the Tri‐M sequence being open. Ultimately, even at 6 months after viral injection, the low labeling density (using the Dre/Flp/Cre combination) in Tri‐M2 mice remained approximately 1‰, still meeting the requirements for single‐cell‐level research.

A limitation of the Tri‑M system is its experimental complexity, encompassing both materials and procedures. For simple fluorescence‑based sparse labeling of specific cell types, at least two mouse lines (Cre driver and Tri‑M) need to be crossed, and site‑directed viral injection is required. To achieve graded sparsity, at least three viral vectors carrying different recombinases must be prepared, with additional vectors needed for functional studies. Thus, the Tri‑M system demands meticulous experimental handling and entails relatively high financial and time costs.

## 4 Materials and Methods

### 4.1 Mice

The Tri‐M1 and Tri‐M2 mouse lines were produced by Gempharmatech Co., Ltd, in which the pCAG‐Tri‐M‐tdTomato‐P2A‐tTA2‐WPRE and pTRE2‐Tri‐M‐tdTomato‐P2A‐tTA2‐WPRE constructs were inserted into the *Rosa26* and *TIGRE* loci, respectively, using CRISPR/Cas9. The NEX‐Cre [49, 50] mice were obtained from Dr. Man Jiang's laboratory at Huazhong University of Science and Technology. Calb1‐Cre [51] (Stock No: 028532), PV‐Cre [52] (Stock No: 008069), ChAT‐Cre [53] (Stock No: 006410), and Aldh1l1‐CreERT2 [54] mice (Stock No: 029655) were purchased from the Jackson Laboratory. Mice were housed under identical conditions with a 12 h light/dark cycle (lights on from 8:00 to 20:00) at a constant temperature of 21 ± 1 °C and humidity of 50 ± 5% [55]. Single‐heterozygous Tri‐M mice were crossed with Cre driver mice for breeding, and the resulting double‐heterozygous offspring (Tri‐M::Cre) were used for experimentation at 90 days of age. The Tri‐M1 and Tri‐M2 mouse lines are available from the corresponding author upon reasonable request.

### 4.2 Viral Injection

Mice were anesthetized with intraperitoneal injection of tribromoethanol and secured in a stereotaxic frame (RWD Life Science). Viral vectors (300 nL) were delivered to target brain regions using a microsyringe pump (Nanoliter 2010 Injector, WPI) controlled by a Micro4 controller (WPI) at an infusion rate of 50 nL/min [56]. Stereotaxic coordinates for each injection target were as follows: VTA (AP: − 3.0 mm, ML: ± 0.5 mm, DV: − 4.5 mm); DG (AP: − 1.9 mm, ML: ± 1.25 mm, DV: − 2.0 mm); visual cortex (AP: − 2.45 mm, ML: ± 2.5 mm, DV: − 0.4 mm); basal forebrain (AP: + 0.6 mm, ML: ± 0.15 mm, DV: − 4.5 mm); CA1 (AP: − 1.7 mm, ML: ± 1.2 mm, DV: − 1.2 mm); sensory cortex (AP: − 1.5 mm, ML: ± 1.8 mm, DV: − 0.5 mm). All recombination adeno‐associated viruses (rAAVs) used in this study were generated by Taitool Bioscience Co., Ltd., including rAAV2‐retro‐EF1a‐DIO‐EGFP‐P2A‐VCre‐WPRE (1.17 × 10^13^ vg/mL), rAAV2‐retro‐EF1a‐DIO‐EGFP‐P2A‐Flp‐P2A‐SCre‐WPRE (1.15 × 10^13^ vg/mL), rAAV2‐retro‐EF1a‐DIO‐EGFP‐P2A‐Flp‐P2A‐Dre‐WPRE (1.06 × 10^13^ vg/mL), rAAV2/9‐EF1a‐DIO‐EGFP‐P2A‐VCre‐WPRE (7.58 × 10^12^ vg/mL), rAAV2/9‐EF1a‐DIO‐EGFP‐P2A‐Flp‐P2A‐SCre‐WPRE (4.61 × 10^12^ vg/mL), rAAV2/9‐EF1a‐DIO‐EGFP‐P2A‐Flp‐P2A‐Dre‐WPRE (7.38 × 10^12^ vg/mL), rAAV2‐retro‐EF1a‐DIO‐EBFP‐VCre‐WPRE (1.12 × 10^13^ vg/mL), and rAAV2/9‐pTRE‐ChR2‐EYFP‐WPRE (7.62 × 10^12^ vg/mL).

### 4.3 Tamoxifen Induction

Tamoxifen (MedChemExpress, Cat# HY‐13757A) was dissolved in corn oil (Sigma–Aldrich, Cat# C8267) to prepare a 20 mg/mL working solution. The mixture was placed on a shaker at 37 °C and incubated overnight with gentle agitation, protected from light, to ensure complete dissolution. 1 day after the viral injection, Aldh1l1–CreERT2::Tri‐M1 mice were induced via intraperitoneal injection at a dosage of 100 mg/kg body weight, administered once daily for four consecutive days. 15 days after the final injection, mice were perfused, and brain tissues were harvested for subsequent analysis. All tamoxifen solutions were freshly prepared immediately before use.

### 4.4 Immunofluorescence

Mice were deeply anesthetized with intraperitoneal injection of tribromoethanol and transcardially perfused with ice‐cold phosphate‐buffered saline (PBS) followed by 4% paraformaldehyde (PFA) in PBS. Brains were rapidly dissected, post‐fixed overnight in 4% PFA at 4 °C, and cryoprotected in 30% sucrose in PBS for at least 48 h at 4 °C. Serial coronal sections (30 µm thickness) were cut using a cryostat (CryoStar NX50, Thermo Fisher Scientific) and stored in PBS at 4 °C until staining. For immunofluorescence staining, free‐floating sections were washed three times with PBS containing 0.1% Triton X‐100, blocked with 5% bovine serum albumin (BSA) in PBS for 1 h at room temperature, and incubated with primary antibodies diluted in PBS containing 3% BSA for 36‐48 h at 4 °C. The following primary antibodies were used: chicken anti‐PV (1:500, SYSY, 195006), goat anti‐ChAT (1:500, Millipore, AB144P), and chicken anti‐GFAP (1:500, Invitrogen, PA1‐10004). After washing, sections were incubated with species‐appropriate Alexa Fluor‐conjugated secondary antibodies (1:300, Invitrogen) diluted in PBS containing 3% BSA for 2 h at room temperature, washed again, and counterstained with DAPI (1:1000, Sigma, D9542) or fluorescent Nissl stains (NeuroTrace, 1:500, Thermo, N21483). Fluorescence images were acquired using a confocal laser‐scanning microscope (FV3000, Olympus) and a slide scanner (SLIDEVIEW VS200, Olympus). Sparse labeling efficiency was quantified using ImageJ software (version 1.8.0), defined as the percentage of tdTomato^+^ cells relative to EGFP^+^ cells within specific regions. For each mouse, 3 to 5 sections with Tri‐M1 labeling and at least 5 sections with Tri‐M2 labeling were analyzed to generate a single value, and five mice were used per group.

### 4.5 Electrophysiology

Sagittal sensory cortex slices from NEX‐Cre::Tri‐M1 mice were prepared 15 days after viral injection (rAAV2‐retro‐DIO‐EBFP‐VCre to VTA and rAAV2/9‐pTRE‐ChR2‐EYFP to sensory cortex), and whole‐cell patch‐clamp recordings were performed on EYFP‐labeled pyramidal neurons in L5 of the sensory cortex. In detail, slices containing the sensory cortex were prepared and transferred to a holding chamber filled with artificial cerebrospinal fluid (ACSF in mM: 124 NaCl, 3 KCl, 26 NaHCO_3_, 1.2 MgCl_2_, 1.25 NaH_2_PO_4_∙2H_2_O, 10 C_6_H_12_O_6_, and 2 CaCl_2_ at pH 7.4, 305 mOsm) at 32 °C for 30 min. The temperature was then maintained at 22 °C for 60 min. Slices were transferred to a recording chamber, which was continuously perfused with oxygenated ACSF (2 mL/min) at 22 °C. Neurons were visualized under a fluorescent infrared‐phase‐contrast (IR‐DIC) Axioskop 2FS upright microscope equipped with a Hamamatsu C2400‐07E infrared camera. Whole‐cell patch‐clamp recordings were performed with intracellular solution containing the following (in mM): 120 CsCH_3_SO_3_, 20 CsCl, 4 Nacl, 10 Hepes, 4 Mg‐ATP, 3 QX314, 0.05 EGTA, 0.2 Na‐GTP. Action potentials were recorded by delivery of blue laser light (473 nm light at an intensity of ∼5 mW, DPSS laser, Inper Co., Ltd., Hangzhou, China) onto the soma of sensory cortex ChR2‐EYFP neurons. Light stimulation consisted of 5 pulses (2 ms for each pulse) at 5 Hz and repeated every 5 s for at least 20 sweeps. Signals were low‐pass‐filtered at 2 kHz and sampled at 10–20 kHz with a Digidata 1440A (Molecular Devices). Throughout recordings, series resistances (Rs) were carefully monitored. Recordings were discarded if the series resistance changed >20% and excluded from data analysis.

### 4.6 Statistical Analysis

All data were presented as mean ± SEM and performed using GraphPad Prism software (version 10.0; GraphPad Software, Inc., La Jolla, CA, USA).

## Author Contributions

**Xinyan Li** and **Youming Lu** conceived and designed the studies and drafted the manuscript. **Xiaomei Tang, Zhenye Hou, Yige Song**, and **Bing Zhang** carried out the experiments, including genotyping, viral injection, and immunostaining. **Yige Song, Changdong Chai, Lanfang Li, Shuo Wang, Ya Zhao, Jinyu Zeng, Yiqing Guo, Fuliang Jiang, Zhigao Xiang, Hao Li**, and **Aodi He** performed imaging and data analysis. **Li Lu** and **Yiqing Guo** conducted electrophysiological recordings.

## Conflicts of Interest

The authors declare no conflicts of interest.

## Ethics Statement

All animal procedures were approved by the Institutional Animal Care and Use Committee of the Animal Center, Huazhong University of Science and Technology, Wuhan, China (Approval No. HUST‐IACUC‐2025‐0047), and were conducted in strict accordance with institutional guidelines.

## Supporting information




**Supporting File**: advs774388‐sup8‐00018‐SuppMat.pdf.

## Data Availability

The data that support the findings of this study are available from the corresponding author upon reasonable request.
