# Supplementary material for: A Novel Sparse Cellular Labeling System with Tunable Gradients and Long‐Term Stability
Source: Adv Sci (Weinh). 2026 Aug 30;13(54):e77438. doi: 10.1002/advs.77438 (PMC13526698; doi:10.1002/advs.77438)
Supplement: Supplementary file 1 [file ADVS-13-e77438-s001.pdf]

Figure S1. Principle of Dre and Flp recognition sites.

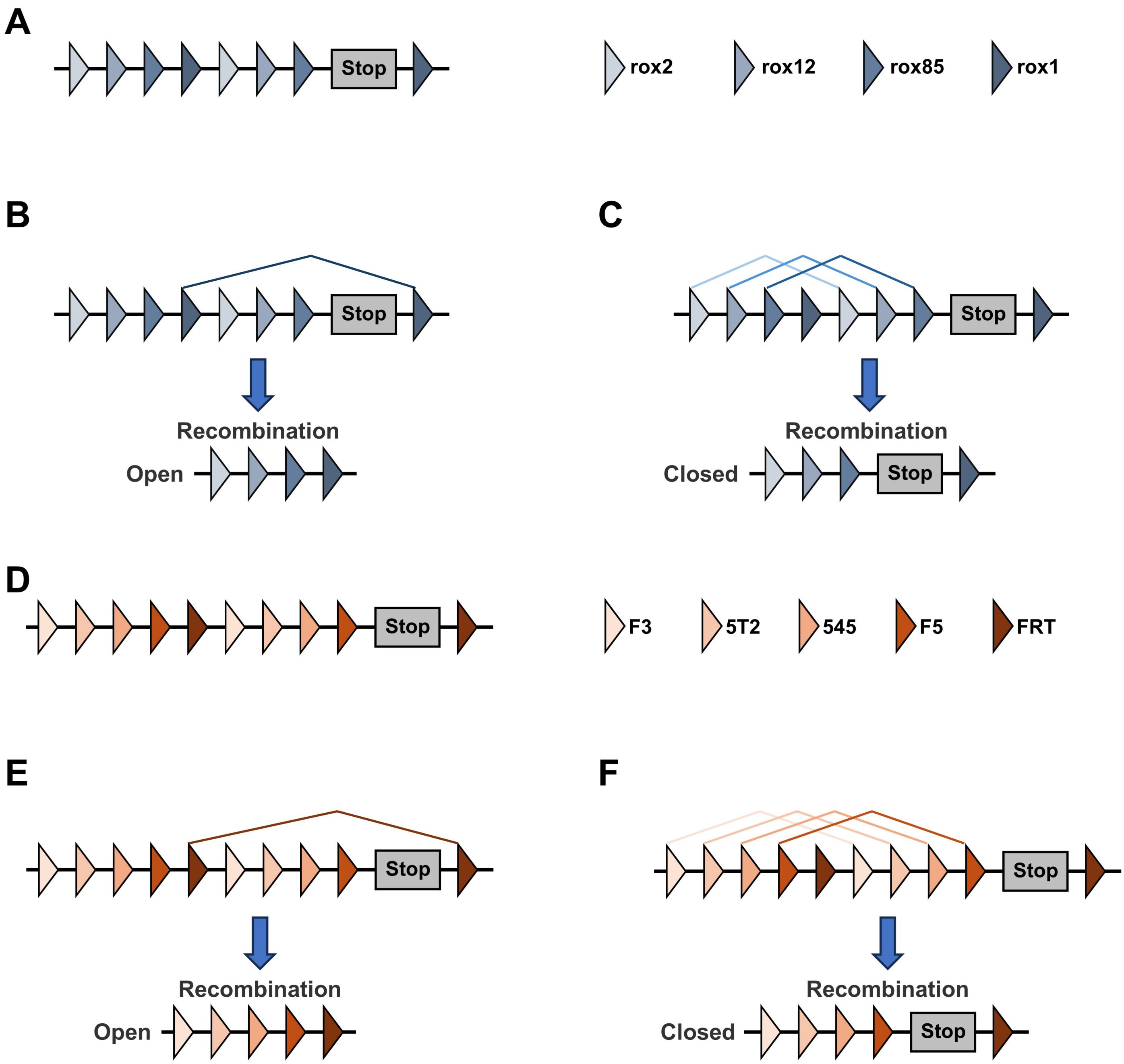

Figure S1. Principle of Dre and Flp recognition sites.

(A-F) Schematic diagrams of the Dre (**A**) and Flp (**D**) recognition sites and their open (**B**, **E**) and closed (**C**, **F**) configurations after recombination.

Figure S2. Projections of graded sparse labeling.

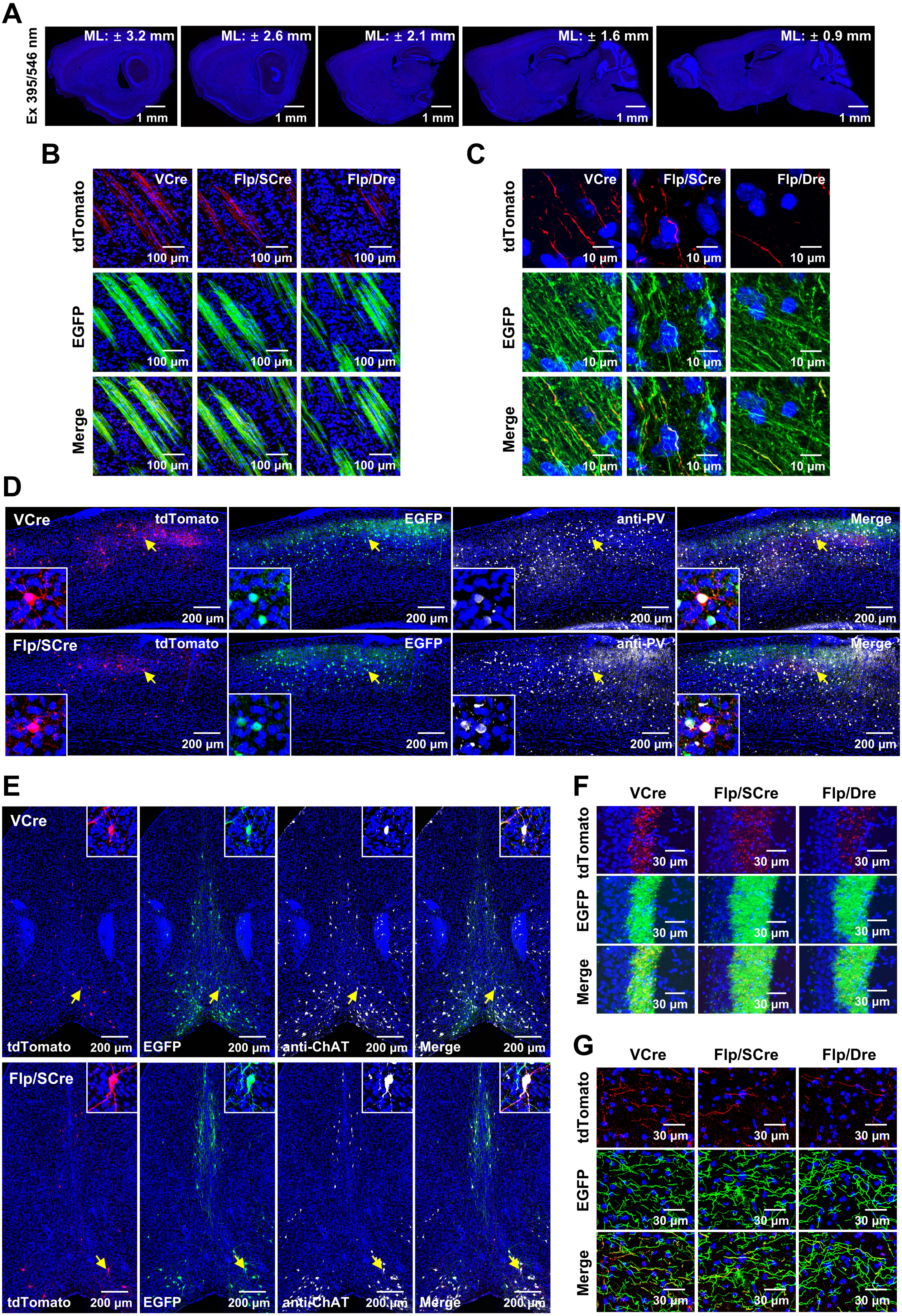

Figure S2. Projections of graded sparse labeling.

(A) Serial brain sections from Tri-M1 mice, showing DAPI (Ex 395 nm) but no detectable cellular signal under Ex 546 nm.

(B, C) Images showing projections in the striatum (**B**) and VTA (**C**) following graded sparse labeling of layer 5 neurons. Related to **Figure 2B-D**.

(D, E) Images showing sparse labeling of PV-positive neurons in the visual cortex (**D**) and cholinergic neurons in the basal forebrain (**E**) with the medium and low sparsity modes of the Tri-M system. Labeling specificity was confirmed by immunostaining with anti-PV and anti-ChAT, respectively. Related to **Figure 3B, C**.

(F) Images showing mossy fibers following graded sparse labeling of granule cells. Related to the boxed regions in **Figure 3A**.

(G) Images showing projections in hippocampus following graded sparse labeling of cholinergic neurons in the basal forebrain. Related to **Figure 3C**.

Figure S3. Changes in labeling sparsity over an extended period in Tri-M2 mice.

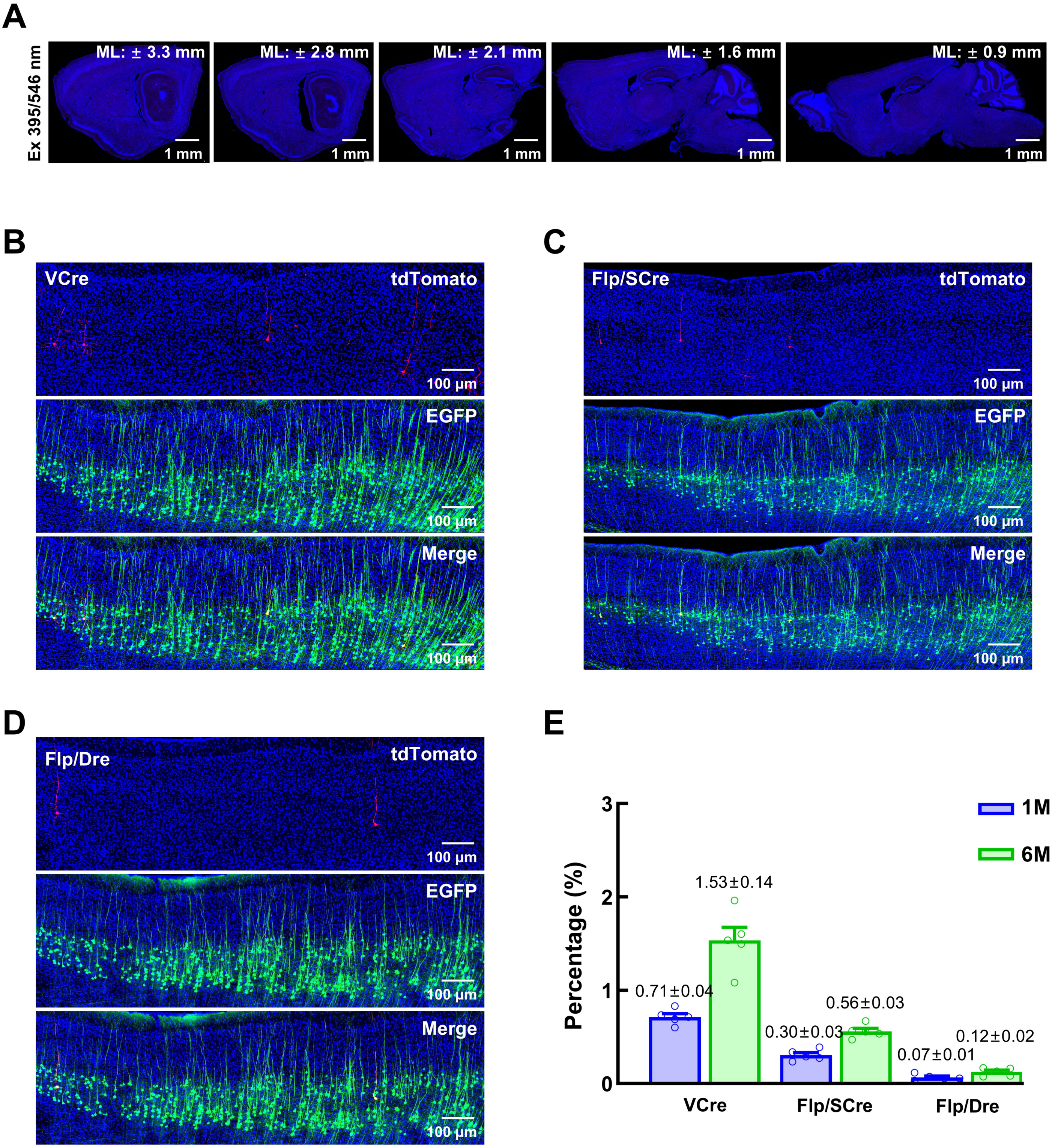

Figure S3. Changes in labeling sparsity over an extended period in Tri-M2 mice.

(A) Serial brain sections from Tri-M2 mice, showing DAPI (Ex 395 nm) but no detectable cellular signal under Ex 546 nm.

(B-D) Images showing graded sparse labeling of L5 excitatory neurons in the cerebral cortex in NEX-Cre::Tri-M2 mice 6 months after viral injection.

(E) A bar graph showing the sparsity of graded labeling of L5 excitatory neurons in the cerebral cortex across the three functional modes of the Tri-M system in NEX-Cre::Tri-M2 mice at 1 month and 6 months after viral injection. Data are presented as mean ± SEM (n = 5 mice per group).

Table S1: The Tri-M sequence

CTCGTGTCCGATAACTGTAATTATCGGACATGATCGGCGCGCTCAATTTCTG

SloxP VloxP

AGAACTGTCATTCTCGGAAATTGACAAGCGCTTAACTTTAAATAATGTCCAT

rox2

TATTTAAAGTTACTGATATCTAACTTTAAATAAGGCCTGTTATTTAAAGTTAC

rox12

GGCGCGCTAACTTTAAATAAGGCCGGTTATTTAAAGTTACAAGCGCTTAACT

rox85

TTAAATAATGCCAATTATTTAAAGTTACTGATATCAGGCGTGCTAGCTAACTT

rox1

TAAATAATGTCCATTATTTAAAGTTACTGATATCAGGCGTGTTTAAACATTAA

rox2

CTTTAAATAAGGCCTGTTATTTAAAGTTACGCGTACGAGGCGTGCTAGCTAA

rox12

CTTTAAATAAGGCCGGTTATTTAAAGTTACAAGCGCTCTGTAAGTCTGCAGA

rox85 PGK poly(A) signal

AATTGATGATCTATTAAACAATAAAGATGTCCACTAAAATGGAAGTTTTTCC
TGTCATACTTTGTTAAGAAGGGTGAGAACAGAGTACCTACATTTTGAATGG
AAGGATTGGAGCTACGGGGGTGGGGGTGGGGTGGGATTAGATAAATGCCT
GCTCTTTACTGAAGGCTCTTTACTATTGCTTTATGATAATGTTTCATAGTTGG
ATATCATAATTTAAACAAGCAAAACCAAATTAAGGGCCAGCTCATTCCTCCC
ACTCATGATCTATAGATCTATAGATCTCTCGTGGGATCATTGTTTTTCTCTTG
ATTCCCACTTTGTGGTTCTAAGTACTGTGGTTTCCAAATGTGTCAGTTTCAT
AGCCTGAAGAACGAGATCAGCAGCCTCTGTTCCACATACACTTCATTCTCA
GTATTGTTTTGCCAAGTTCTAATTCCATCAGAAGCTTGCAGATCTGCGACTC
TAGAGGATCGACTGTGCCTTCTAGTTGCCAGCCATCTGTTGTTTGCCCCTCC

bGH poly(A) signal

CCCGTGCCTTCCTTGACCCTGGAAGGTGCCACTCCCACTGTCCTTTCCTAAT
AAAATGAGGAAATTGCATCGCATTGTCTGAGTAGGTGTCATTCTATTCTGGG
GGGTGGGGTGGGGCAGGACAGCAAGGGGGAGGATTGGGAAGACAATAGC
AGGCATGCTGGGGATGCGGTGGGCTCTATGGGTGCCTTCTAGTTGCCAGCC

bGH poly(A) signal

ATCTGTTGTTTGCCCCTCCCCCGTGCCTTCCTTGACCCTGGAAGGTGCCACT

CCCACTGTCCTTTCCTAATAAAATGAGGAAATTGCATCGCATTGTCTGAGTA
GGTGTCATTCTATTCTGGGGGGTGGGGTGGGGCAGGACAGCAAGGGGGAG
GATTGGGAAGACAATGGCAGGCATGCTGGGGAAACTTGTTTATTGCAGCTT
SV40 poly(A) signal
ATAATGGTTACAAATAAAGCAATAGCATCACAAATTTCACAAATAAAGCATT
TTTTTCACTGCATTCTAGTTGTGGTTTGTCCAAACTCATCAATGTATCTTAAA
CATACGCTCTCCATCAAAACAAAACGAAACAAAACAAACTAGCAAAATAG
pause site
GCTGTCCCCAGTGCAAGTGCAGGTGCCAGAACATTTCTCTAGGCGTGTTTA
AACATTAACTTTAAATAATGCCAATTATTTAAAGTTACGCGTACGCTCGTGTC
rox1

CGATAACTGTAATTATCGGACATGATCGCGTACGGAAGTTCCTATTCTTCAA
SloxP F3

ATAGTATAGGAACTTCCAAGCGCTGAAGTTCCTATTCTCTAGAAGGTATAGG
5T2

AACTTCCGGCGCGCGAAGTTCCTATTCTCTAAAAAGTATAGGAACTTCCAA
545

GCGCTGAAGTTCCTATTCTTCAAAAGGTATAGGAACTTCCTGATATCGAAGT
F5

TCCTATTCTCTAGAAAGTATAGGAACTTCCAAGCGCTAGGCGTGCTAGCGA
FRT

AGTTCCTATTCTTCAAATAGTATAGGAACTTCCAAGCGCTAGGCGTGTTTAA
F3

ACATGAAGTTCCTATTCTCTAGAAGGTATAGGAACTTCCTGATATCAGGCGT
5T2

GCTAGCGAAGTTCCTATTCTCTAAAAAGTATAGGAACTTCCGGCGCGCAGG
545

CGTGTTTAAACATGAAGTTCCTATTCTTCAAAAGGTATAGGAACTTCCAAGC
F5

GCTCTGTAAGTCTGCAGAAATTGATGATCTATTAAACAATAAAGATGTCCAC
PGK poly(A) signal
TAAAATGGAAGTTTTTCCTGTCATACTTTGTTAAGAAGGGTGAGAACAGAG
TACCTACATTTTGAATGGAAGGATTGGAGCTACGGGGGTGGGGGTGGGGTG
GGATTAGATAAATGCCTGCTCTTTACTGAAGGCTCTTTACTATTGCTTTATGA

TAATGTTTCATAGTTGGATATCATAATTTAAACAAGCAAAACCAAATTAAGG
GCCAGCTCATTCCTCCCACTCATGATCTATAGATCTATAGATCTCTCGTGGGA
TCATTGTTTTTCTCTTGATTCCCACTTTGTGGTTCTAAGTACTGTGGTTTCCA
AATGTGTCAGTTTCATAGCCTGAAGAACGAGATCAGCAGCCTCTGTTCCAC
ATACACTTCATTCTCAGTATTGTTTTGCCAAGTTCTAATTCCATCAGAAGTGC
CTTCTAGTTGCCAGCCATCTGTTGTTTGCCCCTCCCCCGTGCCTTCCTTGAC

bGH poly(A) signal

CCTGGAAGGTGCCACTCCCACTGTCCTTTCCTAATAAAATGAGGAAATTGC
ATCGCATTGTCTGAGTAGGTGTCATTCTATTCTGGGGGGTGGGGTGGGGCA
GGACAGCAAGGGGGAGGATTGGGAAGACAATGGCAGGCATGCTGGGGAA
ACTTGTTTATTGCAGCTTATAATGGTTACAAATAAAGCAATAGCATCACAAA

SV40 poly(A) signal

TTTCACAAATAAAGCATTTTTTTCACTGCATTCTAGTTGTGGTTTGTCCAAA
CTCATCAATGTATCTTATCCTCAGGTGCAGGCTGCCTATCAGAAGGTGGTGG
CTGGTGTGGCCAATGCCCTGGCTCACAAATACCACTGAGATCTTTTTCCCTC
TGCCAAAAATTATGGGGACATCATGAAGCCCCTTGAGCATCTGACTTCTGG
CTAATAAAGGAAATTTATTTTCATTGCAATAGTGTGTTGGAATTTTTTGTGTC

β-globin poly(A) signal

TCTCACTCGGAAGGACATATGGGAGGGCAAATCATTTAAAACATCAGAATG
AGTATTTGGTTTAGAGTTTGGCAACATATGCCCATATGCTGGCTGCCATGAA
CAAAGGTTGGCTATAAAGAGGTCATCAGTATATGAAACAGCCCCCTGCTGT
CCATTCCTTATTCCATAGAAAAGCCTTGACTTGAGGTTAGATTTTTTTTATAT
TTTGTTTTGTGTTATTTTTTTCTTTAACATCCCTAAAATTTTCCTTACATGTTT
TACTAGCCAGATTTTTCCTCCTCTCCTGACTACTCCCAGTCATAGCTGTCCC
TCTTCTCTTATGGAGATCAACATACGCTCTCCATCAAAACAAAACGAAACA

pause site

AAACAAACTAGCAAAATAGGCTGTCCCCAGTGCAAGTGCAGGTGCCAGAA
CATTTCTCTAGGCGTGCTAGCGAAGTTCCTATTCTCTAGAAAGTATAGGAAC

FRT

TTCCTGATATCTCAATTTCTGAGAACTGTCATTCTCGGAAATTGACGCGTAC

VloxP

GATAACTTCGTATAGTATACCTTATACGAAGTTATCGGCGCGCATAACTTCGT

loxN

ATAGGATACTTTATACGAAGTTATCAAGCGCTATAACTTCGTATAATGTGTAC

lox2272 lox5171

TATACGAAGTTATCTGATATCATAACTTCGTATAATGTATACTATACGAAGTTA

lox511

TCGCGTACGATAACTTCGTATAGCATACATTATACGAAGTTATCGGCGCGCA

loxP

GGCGTGTTTAAACATATAACTTCGTATAGTATACCTTATACGAAGTTATCTGA

loxN

TATCAGGCGTGCTAGCATAACTTCGTATAGGATACTTTATACGAAGTTATCGC

lox2272

GTACGAGGCGTGTTTAAACATATAACTTCGTATAATGTGTACTATACGAAGT

lox5171

TATCTGATATCAGGCGTGCTAGCATAACTTCGTATAATGTATACTATACGAAG

lox511

TTATCGGCGCGCCTGTAAGTCTGCAGAAATTGATGATCTATTAAACAATAAA

PGK poly(A) signal

GATGTCCACTAAAATGGAAGTTTTTCCTGTCATACTTTGTTAAGAAGGGTGA
GAACAGAGTACCTACATTTTGAATGGAAGGATTGGAGCTACGGGGGTGGG
GGTGGGGTGGGATTAGATAAATGCCTGCTCTTTACTGAAGGCTCTTTACTAT
TGCTTTATGATAATGTTTCATAGTTGGATATCATAATTTAAACAAGCAAAACC
AAATTAAGGGCCAGCTCATTCCTCCCACTCATGATCTATAGATCTATAGATCT
CTCGTGGGATCATTGTTTTTCTCTTGATTCCCACTTTGTGGTTCTAAGTACTG
TGGTTTCCAAATGTGTCAGTTTCATAGCCTGAAGAACGAGATCAGCAGCCT
CTGTTCCACATACACTTCATTCTCAGTATTGTTTTGCCAAGTTCTAATTCCAT
CAGAAGCTTGCAGATCTGCGACTCTAGAGGATCGACTGTGCCTTCTAGTTG

bGH poly(A) signal

CCAGCCATCTGTTGTTTGCCCCTCCCCCGTGCCTTCCTTGACCCTGGAAGG
TGCCACTCCCACTGTCCTTTCCTAATAAAATGAGGAAATTGCATCGCATTGT
CTGAGTAGGTGTCATTCTATTCTGGGGGGTGGGGTGGGGCAGGACAGCAA
GGGGGAGGATTGGGAAGACAATAGCAGGCATGCTGGGGATGCGGTGGGCT
CTATGGCTGCGACTCTAGAGGATCATAATCAGCCATACCACATTTGTAGAGG
TTTTACTTGCTTTAAAAAACGTTTAAACCTCCCACACCTCCCCCTGAACCTG
AAACATAAAATGAATGCAATTGTTGTTGTTAACTTGTTTATTGCAGCTTATAA

SV40 poly(A) signal

TGGTTACAAATAAAGCAATAGCATCACAAATTTCACAAATAAAGCATTTTTT
TCACTGCATTCTAGTTGTGGTTTGTCCAAACTCATCAATGTATCTTATCATGT
CTGGATCTGCGACTCTAGAGGATCATAATCAGCCATACCACATTTGTAGAGG
TTTTACTTGCTTTAAAAAACCTCCCACACCTCCCCCTGAACCTGAAACATA
AAATGAATGCAATTGTTGTTGTTAACTTGTTTATTGCAGCTTATAATGGTTAC

AAATAAAGCAATAGCATCACAAATTTCACAAATAAAGCATTTTTTTCACTGC

SV40 poly(A) signal

ATTCTAGTTGTGGTTTGTCCAAACTCATCAATGTATCTTATCATGTCTGGATC
TGCGACTCTAGAGGATCATAATCAGCCATACCACATTTGTAGAGGTTTTACT
TGCTTTAAAAAACCTCCCACACCTCCCCCTGAACCTGAAACATAAAATGAA
TGCAATTGTTGTTGTTAACTTGTTTATTGCAGCTTATAATGGTTACAAATAAA

SV40 poly(A) signal

GCAATAGCATCACAAATTTCACAAATAAAGCATTTTTTTCACTGCATTCTAG
TTGTGGTTTGTCCAAACTCATCAATGTATCTTATCATGTCTGGATCCCCATCA
AGCTGATAACATACGCTCTCCATCAAAACAAAACGAAACAAAACAAACTA

pause site

GCAAAATAGGCTGTCCCCAGTGCAAGTGCAGGTGCCAGAACATTTCTCTAG
GCGTGTTTAAACATATAACTTCGTATAGCATACATTATACGAAGTTAT

loxP

Table S2: Summary of recombinase recognition sites in the Tri-M system.

Recombinase target sites	References
rox2	PMID: 24908100;
rox12	PMID: 26715092; 40730489
rox85	PMID: 26715092; 40730489
rox1	PMID: 15550568; 24908100
F3	PMID: 7947678; 14723844; 32574559
5T2	PMID: 7947678; 20650281
545	PMID: 3540590; 23866336
F5	PMID: 7947678; 14723844; 24908100
FRT	PMID: 14723844; 24908100; 3540590
loxN	PMID: 19145242; 17972876
lox2272	PMID: 19145242; 21288882; 17972876
lox5171	PMID: 9714735; 14723844
lox511	PMID: 3457367; 11418130
loxP	PMID: 21288882; 12136089; 17972876
SloxP	PMID: 21288882; 21548907
VloxP	PMID: 21288882; 21548907
